# Dynamic models of pneumococcal carriage and the impact of the Heptavalent Pneumococcal Conjugate Vaccine on invasive pneumococcal disease

**DOI:** 10.1186/1471-2334-10-90

**Published:** 2010-04-08

**Authors:** Alessia Melegaro, Yoon Hong Choi, Robert George, W John Edmunds, Elizabeth Miller, Nigel J Gay

**Affiliations:** 1 DONDENA Centre for Research on Social Dynamics, Bocconi University, Via Guglielmo Rontgen, Milan, Italy; 2 Centre for Infections, Health Protection Agency, 61 Colindale Avenue, NW9 5EQ London, UK; 3 Infectious Disease Epidemiology Unit, London School of Hygiene and Tropical Medicine, Keppel Street, London, UK

## Abstract

**Background:**

The 7-valent pneumococcal conjugate vaccine has been introduced in national immunisation programmes of most industrialised countries and recently in two African GAVI eligible countries (Rwanda and The Gambia). However the long term effects of PCV are still unclear, as beneficial direct and herd immunity effects might be countered by serotype replacement.

**Method:**

A dynamic, age-structured, compartmental model of *Streptococcus pneumoniae *transmission was developed to predict the potential impact of PCV7 on the incidence of invasive disease accounting for both herd immunity and serotype replacement effects. The model was parameterised using epidemiological data from England and Wales and pre and post-vaccination surveillance data from the US.

**Results:**

Model projections showed that serotype replacement plays a crucial role in determining the overall effect of a PCV7 vaccination programme and could reduce, negate or outweigh its beneficial impact. However, using the estimate of the competition parameter derived from the US post-vaccination experience, an infant vaccination programme would prevent 39,000 IPD cases in the 20 years after PCV7 introduction in the UK. Adding a catch-up campaign for under 2 or under 5 year olds would provide a further reduction of 1,200 or 3,300 IPD cases respectively, mostly in the first few years of the programme.

**Conclusions:**

This analysis suggests that a PCV vaccination programme would eradicate vaccine serotypes from circulation. However, the increase in carriage of non-vaccine serotypes, and the consequent increase in invasive disease, could reduce, negate or outweigh the benefit. These results are sensitive to changes in the protective effect of the vaccine, and, most importantly, to the level of competition between vaccine and non-vaccine types. The techniques developed here can be used to assess the introduction of vaccination programmes in developing countries and provide the basis for cost-effectiveness analyses.

## Background

Despite the considerable burden of disease caused by *Streptococcus pneumoniae *infection in developing countries, the available 7-valent pneumococcal conjugate vaccine (PCV7) introduced in 2000 in the US and included in a number of European countries routine immunisation schedules [1], has yet to be launched in the majority of low-income settings where it is most needed [2]. If on one hand the high price of the vaccine has been a limiting factor, discussions are also ongoing in regards to the feasibility of the programme as well as its long term effectiveness. Though in fact the vaccine has been shown to be effective against invasive and non invasive disease [3-8], the current licensed product contains seven serotypes only (14, 6B, 19F, 23F, 4, 9V, 18C) and it cannot be expected to provide any protection against carriage or disease caused by most other serotypes. On the contrary, evidence from several countries suggests that serotype replacement in carriage generates as a consequence of vaccination [9-12]. An increase in carriage prevalence of serotypes not contained in the vaccine as well as in disease has now been reported suggesting that replacement in carriage may also lead to replacement in disease [13-16].

While surveillance systems can and should closely monitor country-specific trends in the post vaccination era, there is the need to investigate the potential scenarios that the longer term effects of these interventions might produce and to evaluate the economic acceptability of such interventions considering medium to longer term consequences such as herd immunity and serotype replacement.

Transmission dynamic models have now been extensively used to describe the dynamics of infectious diseases [17] and to predict the effects of vaccination against many childhood infections [18-24]. These models are needed to evaluate the amount of herd immunity that can be generated by these programs [25]. Also, by describing the natural history of the infection, they can provide insights on the magnitude of other indirect effects, such as serotype replacement, and on the aspects that need to be considered when evaluating the overall impact of these mechanisms. Here we use population-based data to develop, parameterise and apply an age-structured transmission dynamic model [26] and to explore the impact of different PCV7 vaccination strategies on carriage of vaccine- and non-vaccine pneumococcal serotypes (VT and NVT) and on invasive pneumococcal disease (IPD). This model differentiates from previous more theoretical work [27,28] by the inclusion of an age structure, heterogeneous mixing and the calculation of IPD rates. An age-structured model was considered essential to assess the public health and economic impact of alternative vaccination policies, since the risks of pneumococcal carriage and disease are highly correlated with age, and vaccination programs are targeted at specific age groups (routine vaccination, booster doses, and catch-up campaigns). Our work is also different from other recent and more applied models which looked at the effects of infant vaccination on the epidemiology of *S. pneumoniae *infection, by using pre and post invasive disease surveillance data from, respectively, Australia and the US [29,30]. Neither of these two studies, in fact, looked at the impact of vaccination on the ecology of the bacterium and, in particular, on the possibility that non-vaccine serotypes will replace serotypes eliminated by the vaccine. This phenomenon is currently being observed in England and Wales post vaccine introduction http://www.hpa.org.uk and it is having major implications for the overall impact of the programme.

The model presented here generates projections for England and Wales and evaluates the policies that were under consideration at the Department of Health for the UK at the time of introduction of PCV7. However, future changes to the vaccine formulations and/or schedule can be considered with the current model and it can also be adapted to look at other countries programs. Mathematical models can provide insights on the overall impacts produced by alternative public health interventions and should be used by policy makers as an additional tool to inform their decision.

## Methods

### Data

The following three distinctive datasets were used to parameterise the model:

• pneumococcal carriage prevalence data from England and Wales [31] to estimate the pre-vaccination steady state force of infection

• the national enhanced surveillance of IPD in England and Wales [32] to derive the *case:carrier *ratio (i.e. the proportion of infection that progress into a disease state)

• the Active Bacterial Core Surveillance data (ABC) [33] which, by providing unique at the time of the analysis pre and post vaccination data allowed the estimation of key model parameters such as degree and duration of vaccine protection and competition between vaccine and non-vaccine serotypes.

In the following a brief description of the three datasets is given.

#### England &Wales longitudinal carriage data

**Figure 1:**
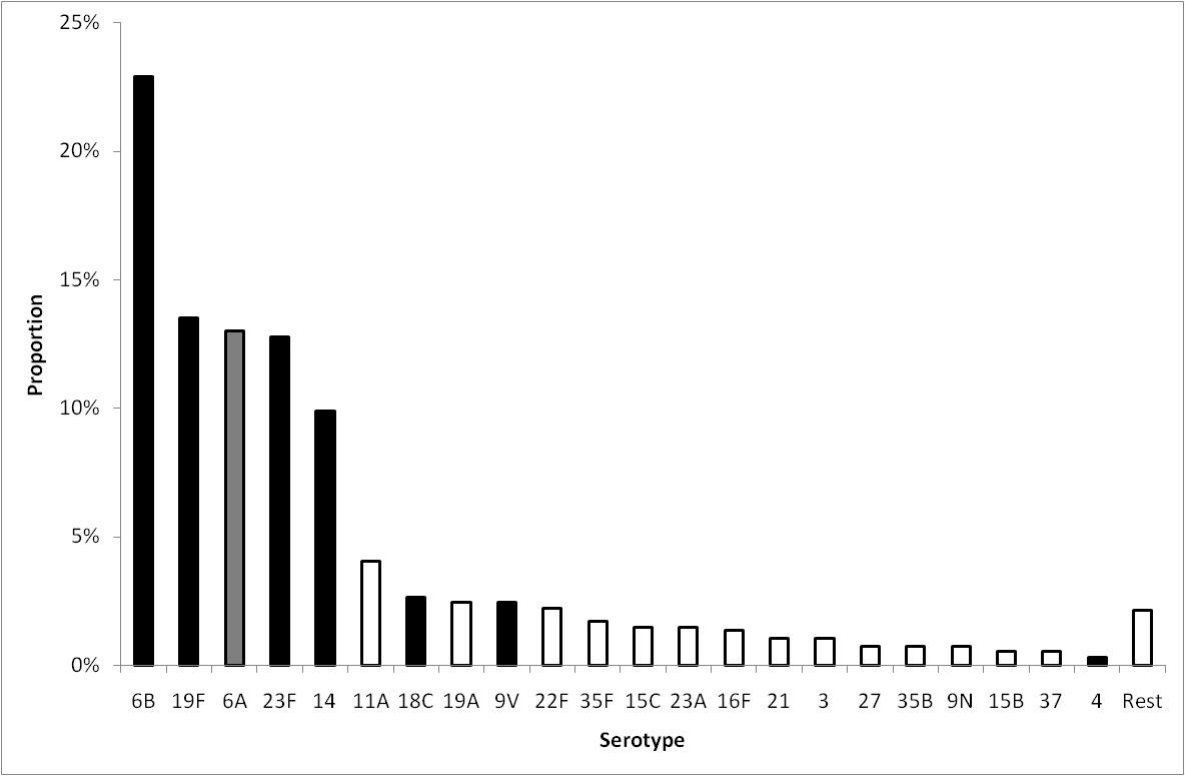
**Serotype distribution of pneumococcal carriage, England and Wales**. Serotype distribution of pneumococcal carriage from a longitudinal study carried out by collecting nasopharyngeal swabs every month from 489 individuals including 121 pre-school children (<3 yrs.) for a period of 10 months, from October 2001 to July 2002 in England & Wales [31,34]. Black bars are serotypes included in PCV7 and white bars are serotypes not included in PCV7. One serotype, 6A, is included in the vaccine serotype group due to evidence of cross-protection.

A longitudinal study was carried out by collecting nasopharyngeal swabs every month from 489 individuals including 121 pre-school children (<3 yrs.) for a period of 10 months, from October 2001 to July 2002 in England and Wales [31,34]. Swabs were cultured for pneumococci according to the WHO guidelines [35]. The number of positive pneumococcal swabs was 932 among the 3,753 swabs taken, (25%), and 34 pneumococcal serotypes were identified (Figure 1). Among these serotypes, 6A, 6B, 14, 19F and 23F were the major serotypes identified from positive swabs (75% in children and 52% in adults). The serotypes included in PCV7 and 6A were identified from 80% of positive swabs from children aged under 5 and 67% of those from subjects aged 5 and older.

#### England &Wales national surveillance of invasive pneumococcal disease

**Figure 2:**
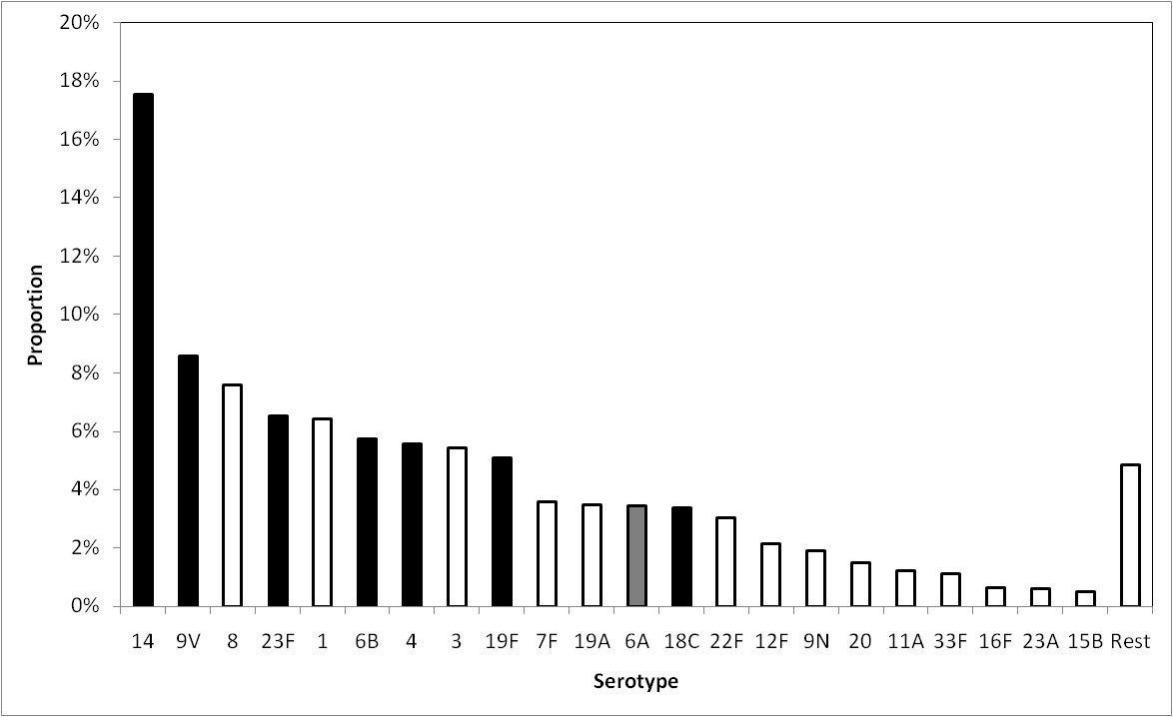
**Serotype distribution of invasive pneumococcal disease cases, England and Wales**. Serotype distribution of invasive pneumococcal disease (IPD) isolates reported to the national enhanced surveillance system in England and Wales. IPD cases in the vaccine serotype group are shown in black bars and IPD cases in the non-vaccine serotype group in white bars. Data shown refer to the years 2004 and 2005.

The annual number of IPD cases was estimated as the average of the overall number of cases that were reported to the reconciled enhanced surveillance system of invasive pneumococcal disease for England & Wales [36,37] and over the 2003/04 and 2004/2005 epidemiological years. This surveillance system is currently the largest national data source on invasive disease caused by *S. pneumoniae *infection and on the isolated serotypes (Figure 2). The average annual number of IPD cases during the pre-vaccination period (between 2003/04 and 2004/05) in England and Wales was 6,184.

#### Active Bacterial Core surveillance of invasive pneumococcal disease

**Figure 3:**
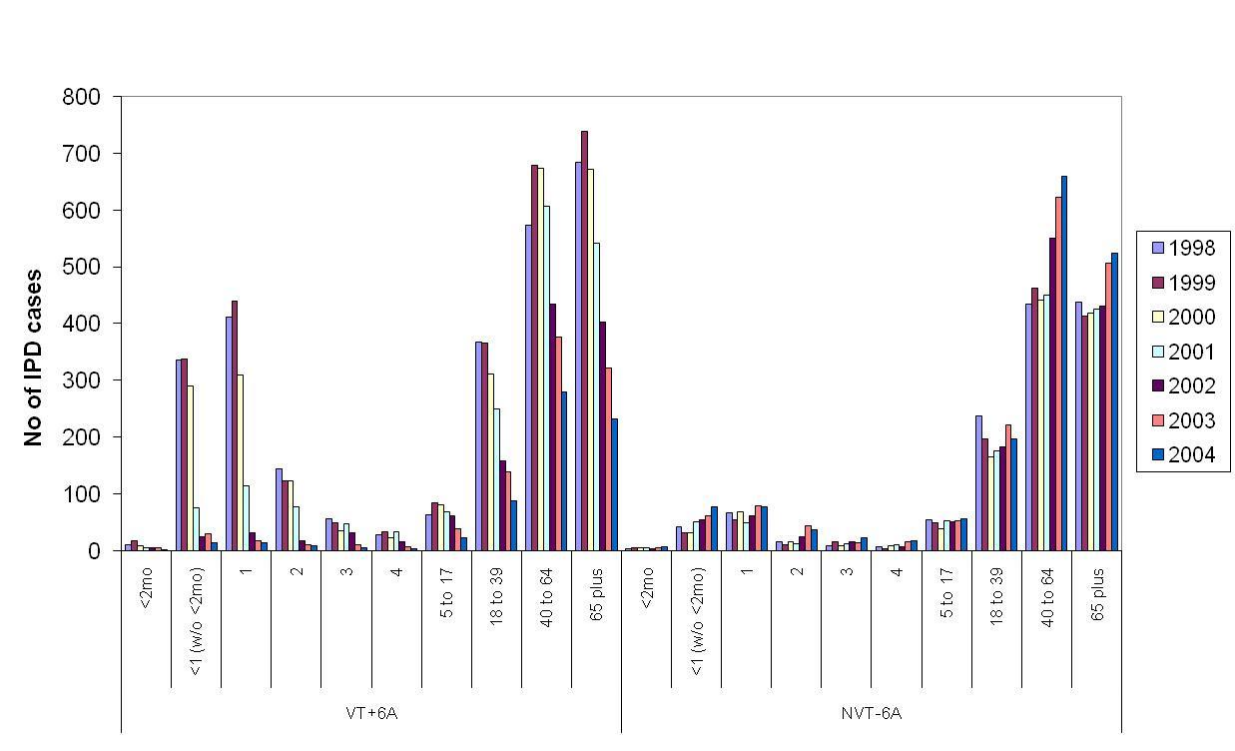
**Pre and post PCV7 vaccination IPD data in the US**. Age distribution of IPD cases belonging to the vaccine serotype group (+6A) and non-vaccine serotype group (-- 6A) in 8 counties included in the Active Bacterial Core Surveillance data in the US between 1998 and 2004.

The Active Bacterial Core Surveillance (ABCs) is an active laboratory and population-based surveillance system for invasive bacterial pathogens of public health importance. From the ABCs system, IPD cases were identified from 8 counties in the US (California, Connecticut, Georgia, Maryland, Minnesota, New York, Oregon and Tennessee). The total population is estimated at 18,041,585 (2004 post-censual estimates) and the total number of IPD cases was 22,365 for the period 1998-2004 [33]. As the PCV7 was introduced in mid 2000, the average number of IPD cases during 1998 and 1999 was used as the pre-vaccination figure and the effects of PCV7 are presented in Figure 3.

### Model

#### Population

The model consists of 100 cohorts of individuals (0, 1, 2, 3,..., 99) each corresponding to one year of age and each of equal size (n_c _= 0.01), with a total stable population (i.e. births equal deaths) which adds up to 1 [26]. Individuals are born into age cohort 0 at the start of the year and live to the age of 99 years, at which point they die. Ageing is thus modelled at the end of each year, with individuals in age group *i *moving up to become age group *i+1*. Each age cohort goes through the transmission cycles according to the infection process outlined in the following paragraph.

#### Structure

A compartmental transmission dynamic model is developed to examine the effects of PCV7 on VT and NVT carriage prevalence and, ultimately, on the overall incidence of IPD. Serotypes 4, 6B, 9V, 14, 18C, 19F, 23F as well as serotype 6A are all included in the VT group. Though 6A is not explicitly included in PCV7, carriage of this serotype is assimilated to the VT one as PCV7 was shown to elicit strong opsonic capacity against serotype 6A [38] and a significant reduction of 6A carriage was observed following vaccination [10,39,40]. In the model, carriage of any of the remaining pneumococcal serotypes is considered a NVT carriage episode.

**Figure 4:**
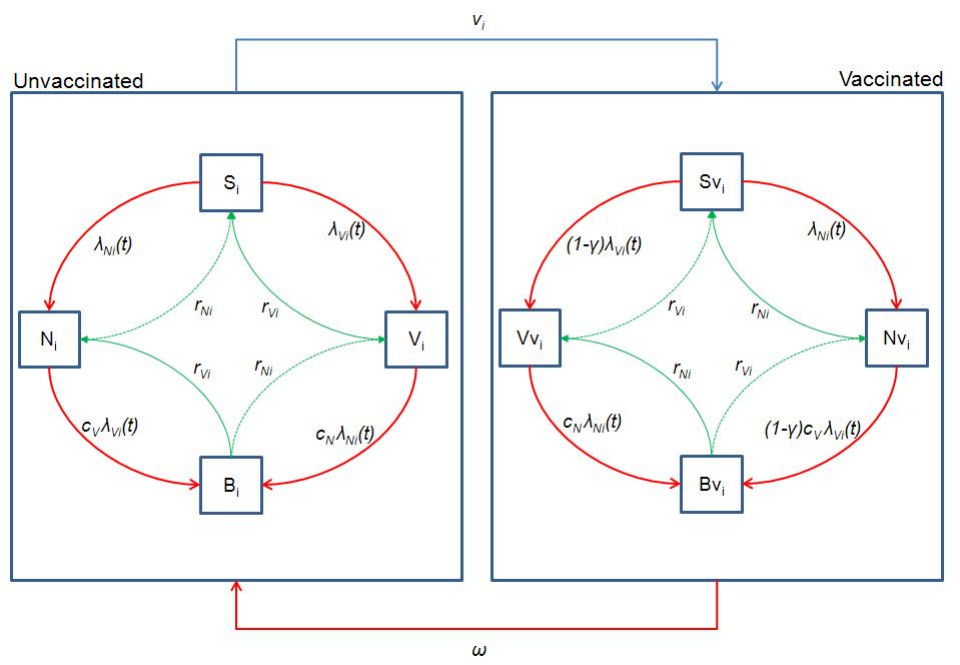
**Model structure**. Flow diagram illustrating the structure of the dynamic model used to assess pneumococcal vaccination. The following states are included: *S*, unvaccinated susceptible; *Sv*, susceptible vaccinated; *V*, unvaccinated carriers of pneumococcal serotypes contained in PCV7 or serotype 6A (vaccine serotype group, VT); *Vv*, vaccinated carriers of pneumococcal serotypes contained in PCV7 or serotype 6A; *N*, unvaccinated carriers of pneumococcal serotypes not contained in PCV7 (non-vaccine serotype group, NVT); *Nv*, vaccinated carriers of pneumococcal serotypes not contained in PCV7; *i*, age; *t*, time; *λ*, force of infection; *γ*, degree of protection against acquisition of VT carriage; *ν*, vaccine coverage; *c*, competition parameters; *ω*, waning rate of vaccine induced protection.

The model consists of a set of ordinary differential equations, which describes the transmission and clearance of carriage with and without vaccination. The model structure is shown in Figure 4 and details of the equations are provided in Appendix 1. A Susceptible-Infected-Susceptible (SIS) type model is chosen (i.e. no immunity after infection) and individuals can get co-infected with one serotype from the VT group and one from the NVT group. Thus, a model that did not consider vaccination would require four compartments: S, non-carriers; V, carriers of VT pneumococci; N, carriers of NVT pneumococci; B, carriers of both VT and NVT pneumococci. To enable consideration of vaccines that provide partial protection against carriage, the model has four additional compartments for individuals that have a degree (i.e. vaccine efficacy against VT carriage acquisition) of vaccine-induced protection (Sv, Vv, Nv, Bv).

##### Infection

Uninfected, unprotected individuals (S) become infected according to the age-specific force of infection of vaccine type (λ_*Vi*_) and non-vaccine type (λ_*Ni*_) pneumococci. Individuals carrying VT become co-colonised at a rate c_*N*_λ_*Ni *_where c_*N *_is the relative susceptibility of an individual colonised with VT to acquiring NVT compared with an uncolonised individual. Similarly NVT carriers become co-infected at a rate c_*V*_λ_*Vi*_. Once infected, individuals can recover from carriage and go back to a susceptible state (V→S or N→S) or, if they were co-colonised, to carriage of a unique type (B→V or B→N). This happens at an age-dependent rate r_*i *_which is the same whether or not the individual was in the colonised state (V or N) or in the co-colonised state (B).

##### Vaccination

Following vaccination, all vaccinated individuals move into the equivalent vaccine-protected compartment according to their carriage status, and gain partial protection against VT acquisition. The force of infection of VT pneumococci, among vaccine-protected individuals, is reduced by a factor (1-*γ*_*i*_) where *γ*_*i *_is the degree of protection. The force of infection for NVT among vaccine-protected individuals is the same as among unprotected individuals. Vaccine-protected individuals may lose their protection and go back to the equivalent unprotected compartment at a waning rate *w *(= 1/δ, where δ is the average duration of protection) that is constant throughout all ages and across all compartments. Vaccination is assumed not to affect infectiousness: vaccinated VT carriers are assumed to be as infectious as unvaccinated VT carriers.

Vaccination programs in the model are implemented as discrete events, rather than as a continuous process at some constant rate: in a routine vaccination program all infants in a cohort are assumed to be vaccinated at the same time according to the vaccination schedule; and in a catch-up campaign individuals in the targeted age group are assumed to be vaccinated simultaneously.

##### Disease

We assume that the risk of disease occurs at the time of carriage acquisition. The proportions of those infected who develop IPD, *Case: Carrier *ratios, depend on their age and the infecting serotype (VT or NVT).

### Parameterisation

#### Recovery rates

The average duration of carriage was estimated using a previously developed modelling framework [41,42] and considering the following age classes: 0-1, 2-4, 5-17 and 18+. Carriage episode was found to be significantly longer in young children (72 days in the 0-1 year of age), and then steadily decreasing with 28 days in the 2-4 years of age, 18 in the 5-17 years and 17 days in the 18+. Age-dependent recovery rates (1/average duration of carriage) were assumed to be the same for VT and NVT (r_Vi _= r_Ni_) [41,43], and also for vaccinated and unvaccinated individuals (Table 1).

**Table 1:** Model parameters

	Base case	Range	Source
Duration of carriage (days):			[41,42]
			
0-1 y	72		
2-4 y	28		
5-17 y	18		
18+ y	17		

c_V_	0.5	0-1	[41]

c_N_	0.85*	0.5-1	*Fitted to US data

ε, mixing pattern	0.87*	0.7-0.95	*Fitted to US data

1/ω, average duration of protection	8.3 y*	5-20 y^#^	*Fitted to US data ^#^95% CI estimated

γ, degree of protection	0.756*	69-84%^#^^	*Fitted to US data ^#^95% CI estimated

^duration and degree of protection are correlated; i.e. short duration is paired with high protection

Transmission parameters of pneumococcal carriage for the base case and ranges used in the sensitivity analyses.

#### Forces of infection

The forces of infection for VT and NVT were age- and time-dependent, varying as a function of the number of vaccinated and unvaccinated carriers in each age class *j *and the rate of effective contacts between individuals in the age class *i *and those in age class *j *(*β*_*ij*_), as follows:

The derivation of the transmission rates β_ij _and the underlying assumptions on the mixing patterns adopted in the model are described in Appendix 2 and follow the techniques originally developed for sexually-transmitted infections [44]. In brief, the contact patterns within and between age groups ranges from fully assortative to proportionate, governed by a single parameter, ε (0≤ε≤1) (ε = 0 proportionate; ε = 1 assortative), which is estimated by fitting model projections to the US post vaccination surveillance data as described below.

#### Mixing and Competition parameters

The introduction of PCV7 for routine use in children aged <5 years in the United States yields valuable information about the impact of vaccination at a population level. We used national surveillance data on the number of cases of invasive disease caused by VT and NVT before and in the four years after the vaccine introduction [45] to estimate mixing pattern and competition parameters. In particular, the impact on VT carriage among the unvaccinated age groups provided information on the mixing patterns in the population (ε). The speed and extent of the increase in NVT disease provided the estimate of c_N _(how much VT carriage protects against acquisition of NVT). As there is little information in this data set on c_V_, we used the value, c_V _= 0.5, as a baseline value, and analysed the sensitivity using the values of 0 and 1. We optimised ε and c_N _by fitting to the incidence of IPD in each age group from the US data by minimizing the Poisson deviance. The reported annual coverage level for 1+ doses in children between 19 and 35 months in 2003 of 86% (Dr Cynthia Whitney, personal communication) was assumed as the baseline annual vaccine coverage of routine vaccination from 2001 onward. Vaccination coverage in the first year of the program was set at 43% for 0 (routine) and 1 (catch-up) year olds as the vaccine was introduced around the middle of the year (July 2000). As the vaccine coverage levels varied each year, the coverage assumed in the model was varied as well, from 80% to 90% with an increment of 2% for the sensitivity analysis.

#### The risk of disease given pneumococcal infection

The age-specific proportions of VT and NVT pneumococcal infections that lead to IPD (case: carrier ratio) were estimated by fitting a model to the age-specific incidence of these diseases attributable to pneumococci as reported from the national enhanced surveillance of pneumococcal disease in the pre-vaccination years [46]. A total of eighteen case: carrier ratios for VT and NVT were estimated for nine age groups, <2 months, 2-11 months, 1-4, 5-14, 15-24, 25-44, 45-64, 65-74, 75+ years.

#### Vaccine associated parameters

We assume that all individuals who received PCV7 move to the vaccine protected group. The average duration and degree of vaccine-induced protection were estimated by fitting the model to the ABCs data from the pre- and post- vaccination US experience. The sensitivity of the model results to these parameter estimates was assessed.

### Modelling PCV7 vaccination

The alternative vaccination strategies investigated here are the ones that were relevant for the UK routine immunisation schedule:

◦ *Strategy 1*: routine vaccination at 2 and 4 months of age, plus a booster dose at 12 m;

◦ *Strategy 2*: Strategy 1 plus catch-up campaign targeting all those under the age of 24 m;

◦ *Strategy 3*: Strategy 1 plus catch-up campaign targeting all those under the age of 60 m.

For each vaccination strategy the model predicted the number of cases of IPD caused by VT and NVT in each age group over time. The coverage of routine and catch-up vaccinations for PCV7 in the UK was assumed to be the same as for the meningococcal serogroup C conjugate vaccine pre-school catch-up (89% for 0 year olds, 84% for 1 year olds, and 76% for the rest) [47]. Catch-up campaigns were implemented at the same time as the routine vaccination was introduced.

### Model analysis

There were three stages to the analysis. First, a steady state pre-vaccination model estimated age-stratified values for the forces of infection and case:carrier ratios for VT and NVT. Second, a dynamic post-vaccination model estimated key vaccine parameters, interaction between VT and NVT and the level of assortativeness of mixing pattern from US surveillance data. Third, the dynamic model was used to assess the impact of alternative vaccination strategies in England and Wales.

The pre-vaccination model was programmed in Excel to estimate forces of infection for VT and NVT and for the following age groups: 0-1, 2-4, 5-9, 10-19, 20-39 and 40-99 using the carriage prevalence data available for England and Wales and a given value for the competition parameter (c_N_). The fully assortative and fully proportionate mixing matrices between the six age-groups were also generated and later used as two extremes in the transmission model. Age-specific case:carrier ratios were then derived fitting the model to the age distribution of IPD cases caused by, respectively, vaccine and non-vaccine serotypes in the pre-vaccination era. The procedure, which required the minimisation of a Poisson deviance using the SOLVER in Excel, was run for both England and Wales and the US to calculate the risk of developing disease when colonised.

The transmission dynamic model was programmed in Berkeley Madonna (R. I. Macey & G. F. Oster, UC Berkeley, CA, USA) and fitted to the pre- and post- vaccination IPD data from the US to estimate degree and duration of protection of the vaccine against invasive disease, and the mixing parameter (ε) for different values of the competition parameter (c_N_). The forces of infection, case:carrier ratios and extreme mixing matrices were updated for each value of c_N_. The estimate of c_N _was derived by finding the value that minimised the deviance.

Once the parameters were generated, the epidemiological model was used to assess the impact of alternative vaccination strategies in England and Wales. The system was solved using the Euler method to integrate ordinary differential equations with fixed time steps of 0.001 years. The model simulated 50 years (five years pre-vaccination and 45 years after vaccination).

## Results

### Model calibration

**Figure 5:**
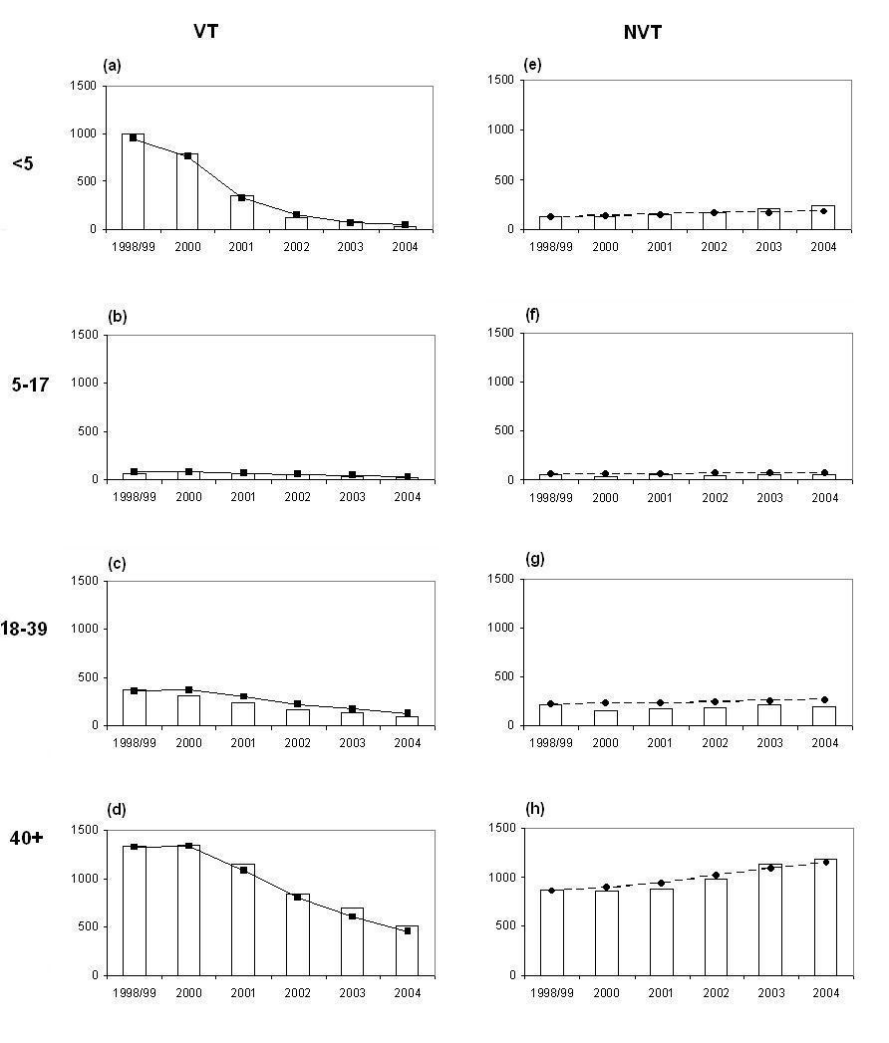
**IPD cases: model projections vs. observed data**. Observed (histograms) and fitted (filled squares and circles with lines) vaccine serotype group (graphs on the left) and non-vaccine serotype group (graphs on the right) IPD cases by age group in 8 counties of USA where the Active Bacterial Core Surveillance data collected the serotyped IPD data between 1998 and 2004.

**Figure 6:**
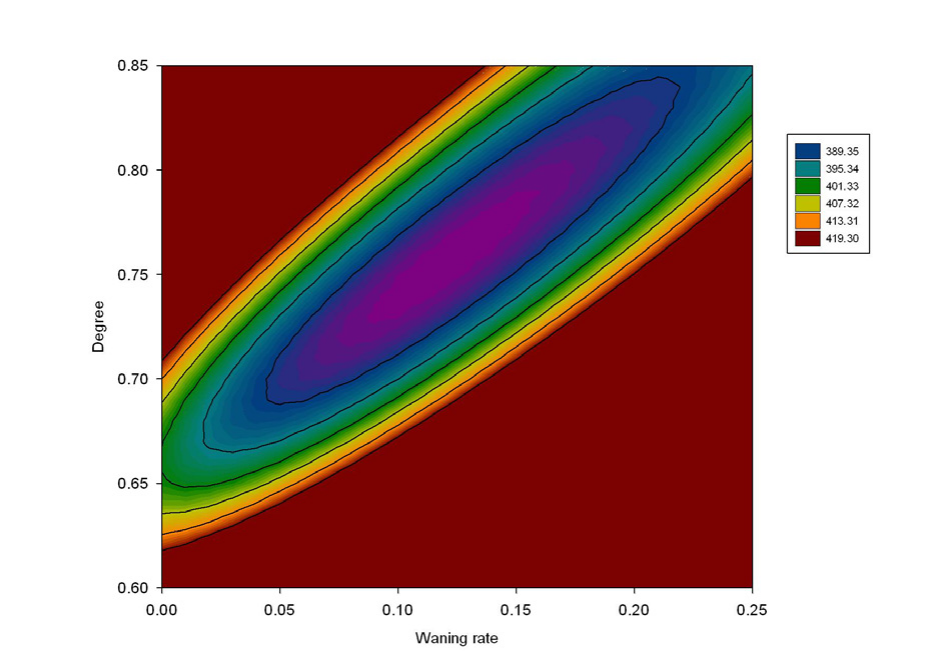
**Estimated degree and duration of protection of the vaccine**. Contour plane of Poisson deviance calculated from fitting the degree and duration of PCV7 protection with the mixing parameter (ε) and competition parameter c_N _fixed to be respectively 0.87 and 0.85.

Graphical comparison between the observed and predicted VT and NVT IPD cases by age group from 1998 to 2004 in the 8 counties covered by the Active Bacterial Core Surveillance is presented in Figure 5. The baseline value of the PCV7 coverage with 1+ doses was assumed to be 86%. The sensitivity of estimates of the degree of vaccine protection and duration of protection to the assumed PCV7 coverage was investigated. Increasing the vaccine coverage assumed may account for some protection derived by individuals who were only partially vaccinated; reducing the vaccine coverage assumed is equivalent to reducing the take of the vaccine to less than 100%. Parameter estimates are presented in Table 2 according to six vaccine coverage values from 80% to 90% and show that increases in the coverage level produced some reduction in the degree and duration of protection as expected. However, for coverage levels within this range, the estimates of the competition and mixing parameters were not sensitive to the assumed vaccination coverage. These results suggest that individuals who were carrying VT were partly protected against NVT acquisition (c_N _= 85%, 15% protection), and that population mixing was closer to assortative than proportionate (ε = 0.87) in line with a recent European contact study [48]. The contour plot presented in Figure 6 describes the 95% confidence area of the degree and duration of the vaccine protection while the mixing and competition parameter were fixed (respectively 0.87 and 0.85).

**Table 2:** Best fitted model parameters estimates

US vaccine coverage	c_N_	Degree	Epsilon	Waning	Duration of PCV7 protection	Deviance
80%	0.86	0.85	0.88	0.12	8.5	381.09
82%	0.85	0.82	0.88	0.12	8.5	381.16
84%	0.86	0.79	0.88	0.12	8.3	382.16
86%	0.85	0.76	0.87	0.12	8.3	383.12
88%	0.85	0.73	0.87	0.12	8.0	384.48
90%	0.85	0.70	0.86	0.13	7.7	386.29

Best fitted results of c_N _(competition parameter), epsilon (mixing parameter), waning (1/duration of vaccine protection), degree of vaccine protection, and Poisson deviance from the optimization according to a 1+ dose of US PCV7 coverage

### Model projections with vaccination

Seven pairs of the degree and duration of PCV7 vaccine protection were chosen within the 95% confidence area (including the best fitted pair with the baseline US PCV7 coverage) and showed that the UK transmission model predictions of IPD changes are insensitive to the assumptions. The fitting results indicated that the higher the degree is the shorter the duration of protection. The baseline parameters used for the following simulations of England and Wales predictions are the ones estimated when fixing the coverage level to 86% (Table 1). Age-specific case:carrier ratios that are used for the UK predictions are shown in Table 3.

**Table 3:** Case:Carrier ratios in the UK

Age group (yrs)	VT	NVT	NVT/VT
0	0.00018	0.00033	1.85
1-4	0.00005	0.00005	0.90
5-14	0.00001	0.00001	1.77
15-24	0.00001	0.00002	2.48
25-44	0.00002	0.00007	3.62
45-64	0.00006	0.00017	2.81
65-74	0.00012	0.00029	2.30
75+	0.00039	0.00064	1.67

Age-specific Case:Carrier ratios for vaccine and non-vaccine serotypes by age groups in the UK

**Figure 7:**
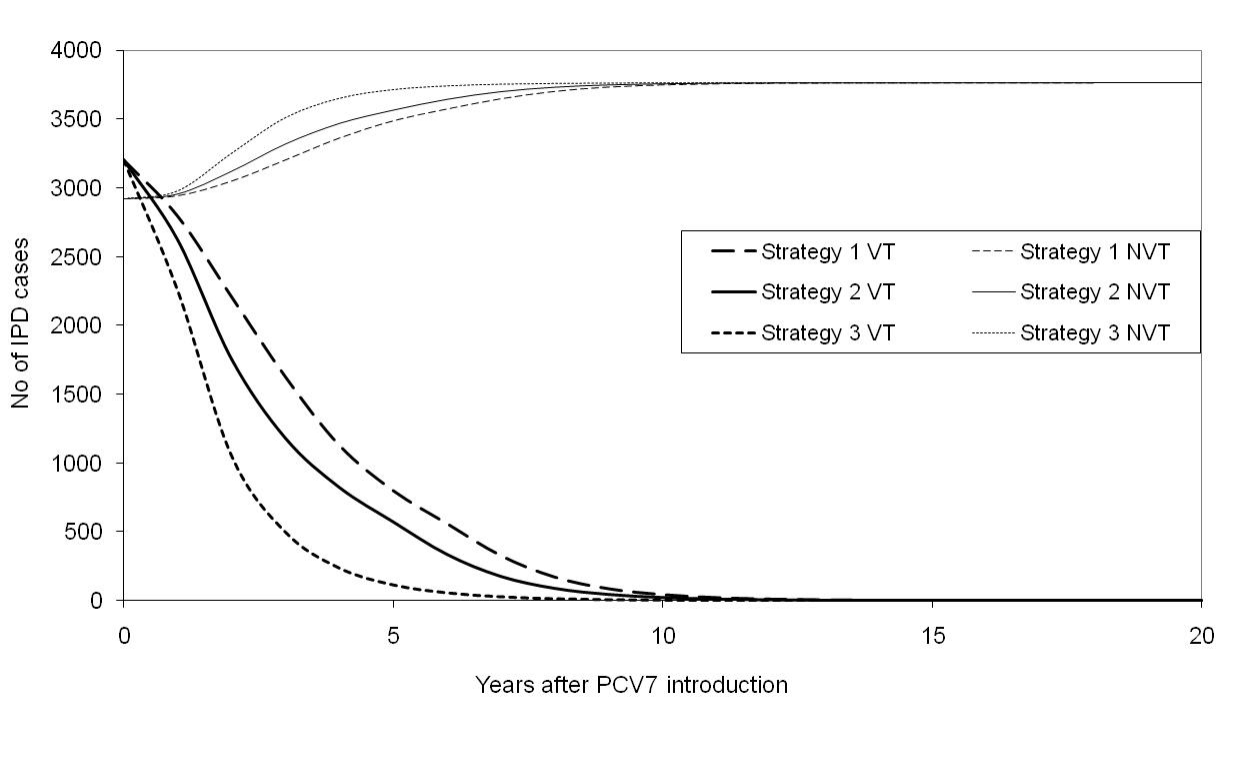
**Model projections for England and Wales**. Estimated annual number of vaccine serotype group and non-vaccine serotype group IPD cases before (Year 0) and after the introduction of PCV7 (Year 1) using three different vaccination strategies in England and Wales.

In the long term, the model predicted that all three strategies had a similar impact and eliminated transmission of VT. Strategy 3, which has the largest catch-up, has the most rapid impact on carriage and IPD, while Strategy 1 with no catch-up was the slowest (Figure 7). The model results presented in Figure 7 also showed an increase in the incidence of NVT carriage and IPD after the introduction of vaccination. The extent of this replacement of VT by NVT is governed by the reduction in VT.

**Figure 8:**
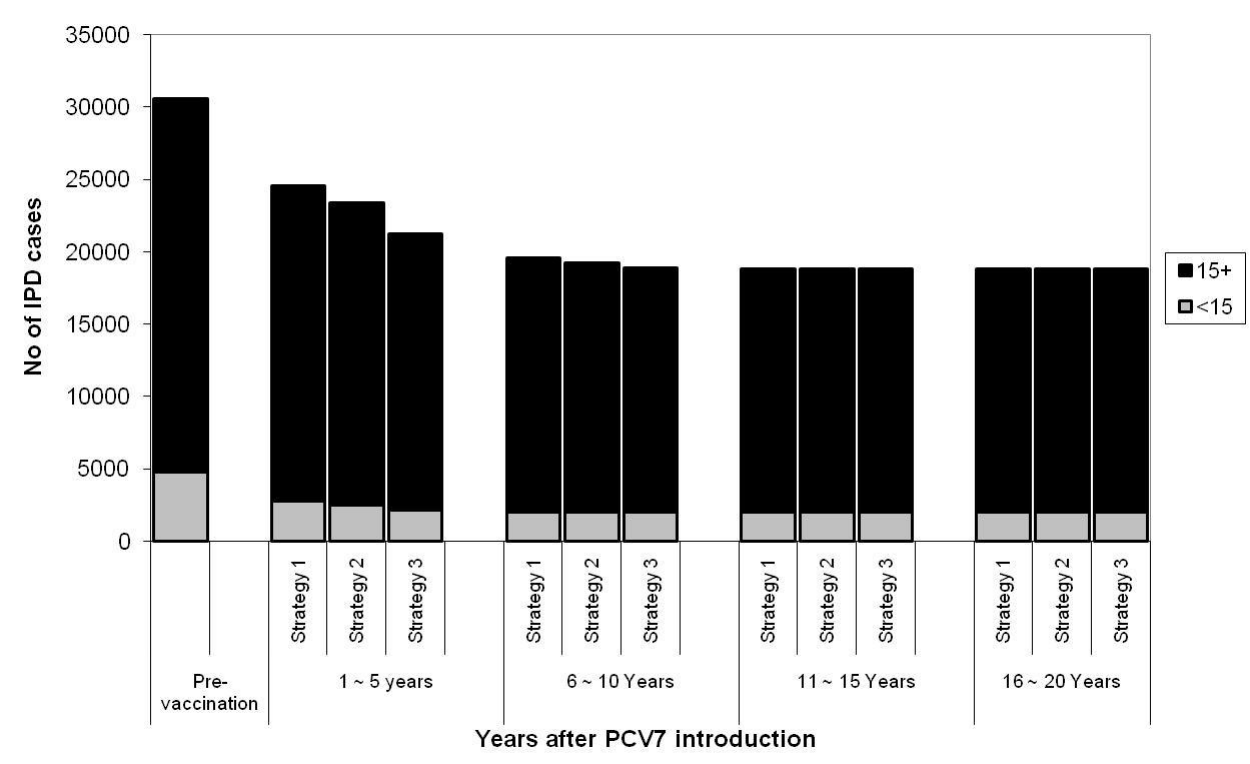
**Estimated number of IPD cases 20 years post vaccination and by age**. Predicted results on the annual number (vaccine (VT) and non-vaccine (NVT) serotype groups) of IPD cases pre-vaccination vs post VT elimination 20 years after the PCV7 introduction by age group in England and Wales.

In the long term, the model predicts a 63% reduction of all IPD cases in children aged less than 5 years old and 35% reduction in the rest of the population, which amounts to the prevention of 2,300 cases of IPD annually after 20 years of the PCV7 introduction with all three vaccine programme strategies. Following the results for the impact on carriage, Strategy 1 is the least effective in terms of disease prevented. The routine vaccination and booster for one year old (Strategy 1) reduces 6000 IPD cases within the first five years of the programme, and an additional catch-up for children up to 23 months of age (Strategy 2) prevents extra 1,200 IPD cases. Extending the catch-up to age 59 month (Strategy 3) prevents further 3,300 IPD cases. The impact of vaccination on the long term incidence of VT and NVT disease by age group and vaccine programme strategies is illustrated in Figure 8.

### Sensitivity analysis

If the vaccine was less protective than assumed here, (i.e. lower duration or degree of protection), the program achieved lower coverage, or the population was less assortative, then transmission of vaccine types may not be eliminated. Extensive sensitivity analysis was conducted and the results are presented in Table 4. However, three main conclusions were drawn. Firstly, a reduction in the duration and/or degree of protection would prevent elimination of VT. However, increasing the values of these parameters (90% degree or 20 years) would not provide much further reduction in disease. Secondly, an increase in the value of the competition parameter c_N _(lower protection from VT carriage) would result in lower replacement of VT by NVT after PCV7 introduction and thus in a small number of IPD cases while a lower competition parameter (i.e. higher protection from VT carriage) would result in more IPD cases than in the pre-vaccination period. Thirdly, lower values of ε (less assortative mixing pattern) would facilitate and hasten elimination of VT transmission with more IPD case reductions. On the contrary, higher values of ε would result in VT continuing to be carried due to less herd immunity effects of the vaccine.

**Table 4:** Sensitivity analysis

		VT IPD	NVT IPD	Total
Baseline		0	3765	3765

V, vaccine coverage	0.89			
	0.5	757	3594	4351
	0.95	0	3765	3765

1/w, Duration of vaccine protection	8.3 years			
	5 Years	788	3570	4358
	20 Years	0	3765	3765

Degree	0.76			
	0.3	1599	3338	4937
	0.9	0	3765	3765

c_V_	0.5			
	0	0	3836	3836
	1	2	3710	3712

c_N_	0.85			
	0	0	9533	9533
	1	0	2921	2921

ε	0.88			
	0	0	3765	3765
	0.95	438	3702	4140

Impact of alternative parameter values on the total number of cases of invasive pneumococcal disease (all ages) caused by vaccine and non-vaccine serotypes (VT and NVT respectively) and predicted by the model in the 20 years following PCV7 introduction under Strategy 1 in England and Wales. Base case parameter values are reported in bold.

## Discussion

The transmission model of *S. pneumoniae *infection presented in this paper enables investigation of the potential effects of infant PCV7 vaccination. It builds on previous *S. pneumoniae *models [27,28] and, by including age-structure, allows investigation of the impact of vaccination on the age-specific incidence of invasive disease.

Simulation results indicated that all of the vaccination strategies considered in this paper were sufficient to eliminate VT transmission in England and Wales with base case values for vaccination coverage and other parameters. Although the model predicts some replacement of VT with NVT, the annual total of IPD cases was reduced from 6,184 to 3,765 (38% reduction) in the long-term (20 years after the PCV7 introduction) and reduction of 39,000 IPD cases accumulated over the period. Catch-up campaigns for children aged under 2 and 5 prevent additional 1,600 and 4,100 IPD cases respectively compared to routine vaccination alone during the first twenty years of the PCV7 program (1,200 and 3,300 cases reduction in the first five years). However, this conclusion is sensitive to vaccine and other model parameters. Small reductions in vaccine coverage, in the duration or degree of protection, from their base case values are sufficient to prevent elimination of VT being achieved. We cannot therefore exclude the possibility that PCV7 vaccination fails to eliminate VT transmission in the UK. On the other hand, NVT dynamics post vaccination are extremely sensitive to the level of competition between VT and NVT, and the model predicted an overall increase of the number of IPD cases pre to post vaccination if the level of protection of VT from NVT acquisition were almost complete.

The competition parameter, c_V _(the relative susceptibility of NVT carriers, compared with uncolonised individuals, to acquiring VT carriage), was assumed to be 50% since there is little information from post-vaccination surveillance on the protective effect of NVT carriage on VT acquisition. Sensitivity analysis suggested that the reduction in annual IPD cases after 10 years of PCV7 introduction is not sensitive to this parameter, with a maximum difference of 1% between scenarios (c_V _= 0 or = 100%). On the other hand, the degree of serotype replacement is sensitive to c_N _(the relative susceptibility of VT carriers, compared with uncolonised individuals, to acquiring NVT carriage), which was estimated by fitting to the US post vaccination surveillance data. The sensitivity analysis indicated that the lower this value, the more replacement of VT by NVT will occur after the introduction of PCV7 in the community. Based on the UK surveillance after the PCV7 introduction, the replacement appears greater than the replacement in the US http://www.hpa.org.uk and the cause of this difference is currently being investigated. Potential contributing factors include differences in secular trends in prevalence and antimicrobial sensitivity of specific serotypes at the time of introduction [49], differences in sensitivity of the respective surveillance systems, or differences in clinical practice post PCV introduction.

The mechanism of competition incorporated in the model may influence the projected outcomes [50]. Here, the mechanism assumed was of a reduction of the acquisition rate of other serotypes when already colonised. This mechanism is supported by a recent Danish longitudinal study of pneumococcal infection [51], which preferred it to a mechanism of competition through an increase in the clearance rate for co-colonised individuals. Our exploration of alternative hypotheses for competition is limited by a lack of co-colonisation data. Clearly, more work is needed in this area and laboratory techniques that enable detection of carriage of multiple serotypes will facilitate studies of the interactions between different pneumococcal serotypes.

The mixing pattern determines herd immunity effects of PCV7 vaccination among the unvaccinated age groups. From the impact observed in the US experience, we estimated that the mixing between individuals is more assortative than proportionate. Thus, similarly to other infections [52], contacts with others in the same age group play an important role in the transmission of pneumococcal carriage. Population mixing patterns are influential parameters in transmission models. True mixing patterns are unknown, and model parameters are often estimated from the pre-vaccination epidemiology after making arbitrary assumptions about the structure of contacts [17]. Here, we use post vaccination herd immunity effects to inform the level of assortativeness of mixing. Alternative approaches to measure age-specific contact patterns directly through diary-based methods are currently being explored using recently collected contact pattern data [53-56].

The relationship between acquisition of carriage and onset of invasive and non-invasive disease is not well understood. Here we assume that the risk of pneumococcal disease occurs at the time of acquisition of carriage, with age- and type-specific proportions developing IPD. An alternative assumption is that this risk is spread over the entire duration of carriage. We do not envisage that adopting this latter mechanism would markedly affect our results, especially as we have assumed that the duration of carriage is the same for both vaccine and non-vaccine types.

One critical aspect of the current work is that the model categorises pneumococcal serotypes as either vaccine-type (including 6A) or non-vaccine type and assumes that the characteristics are homogeneous within each of these two categories. Firstly, the inclusion of serotype 6A in the vaccine group was implemented due to existing evidence that the 7-valent pneumococcal conjugate vaccine induces cross-protection against 6A [14,57]. However, the newly identified serotypes 6C and 6D [58,59], which do not benefit from cross protection [58,60-62] should be excluded from the vaccine group in future modelling work. Secondly, there is considerable heterogeneity between individual serotypes in, for example, their transmissibility, duration of infection, ability to co-colonise, ability to prevent co-colonization with other serotypes, and potential to cause disease [63-65]. All these factors may influence the response of individual serotypes and the pneumococcal population as a whole to the introduction of infant PCV7 vaccination. Studying these effects would require an individual-based model in order to incorporate many circulating serotypes. Within such a framework it would also be possible to begin to investigate the impact of acquired immunity on pneumococcal transmission by incorporating a mechanism for generating type-specific and/or type-independent immunity [66-69].

The impact of pneumococcal vaccination on non-invasive diseases such as Acute Otitis Media (AOM) and Community Acquired Pneumonia (CAP) is not considered in this paper although some level of protection has been reported against these conditions in both developed [4,70] and developing countries [5,71]. Although an individual case of non-invasive pneumococcal disease (NIPD) is generally less severe than IPD, the economic cost of NIPD is higher than that of IPD due to the much greater number of cases in the community. Also, CAP is one of the leading causes of childhood mortality in developing countries, and hence, understanding the impact of pneumococcal vaccine on NIPDs is essential to estimate the overall cost-effectiveness of the program in these settings [72]. The impact of PCV7 vaccination programs on the incidence of pneumococcal AOM and CAP or even on the prevalence of carriage in the general population is currently not well documented. Whilst the capacity of different serotypes to cause AOM is expected to be similar, there are large differences in their risk of causing IPD: some NVT serotypes are very unlikely to cause IPD. The change in prevalence of NVT serotypes that commonly cause IPD can be inferred from the IPD data, but the change in prevalence of NVT serotypes that rarely cause IPD is not observed. The impact on NIPD may not be in line with the effect on IPD. Parameterising models to investigate the effect of the vaccine on AOM or CAP in the community requires more knowledge of the impact of vaccination programs on carriage and NIPD.

The outputs of any model are limited by the validity of the model assumptions and accuracy of the parameter estimates. In this study, considerable attention was paid to estimating appropriate parameter values and to assessing the sensitivity of the results to these values. Data from the longitudinal study in UK families were used to derive the initial prevalence of carriage, recovery rates and the forces of infection. Surveillance data on IPD cases pre- and post- PCV7 introduction in the US were used to estimate vaccine parameters, mixing patterns and to infer the degree of competition between VT and NVT. Moreover, the model developed here may also be useful for other countries considering the introduction of the pneumococcal conjugate vaccine and to estimate effects of alternative vaccine strategies. In future, these models will be improved by refining parameter estimates using the observed effects of the UK programme. The UK experience with the Hib and MenC vaccines [73-75] showed that the post-vaccine era may be far from straightforward and that continued monitoring the duration of vaccine protection and the effects of vaccination on carriage may be critical to the success of the pneumococcal conjugate vaccine program.

## Conclusion

The effect of PCV7 introduction on the burden of IPD is assessed using an age structured dynamic model which was parameterised with UK and US data. Model projections show the importance of considering the existence of competition between serotypes contained and not contained in the vaccine formulation in order to being able to realistically predict possible scenarios. The current model will be extended to incorporate non invasive pneumococcal disease and will form the basis for cost-effectiveness analysis for alternative vaccination programmes, especially in developing countries where the burden of pneumococcal disease is extremely high and the financial resources are limited.

## Competing interests

The authors declare that they have no competing interests.

## Authors' contributions

AM conceived the work, developed the programme and produced the initial draft of the manuscript. NJG contributed to the implementation of the model. YHC extended the programme, contributed to the drafting of the manuscript and performed final simulations. WJE and EM contributed to the interpretation of the results. RG provided the carriage data and contributed to the interpretation of the results. All the authors have read and agreed on the final manuscript.

## Appendices

### A.1. Dynamic model structure in equations

Where i is the age group, ν_i _represents the vaccine coverage in age group i, *π*_i_(t) is a function, describing the vaccination program in cohort *i *to occur at certain time as scheduled, r_Vi _and r_Ni _are, respectively, the proportion that recover from VT and NVT infection, ω is the rate of waning of vaccine-induced protection, *c*_*N *_(*c*_*V*_) represent the relative susceptibility of VT (NVT) carriers, compared with uncolonised individuals, to acquiring NVT (VT) carriage (i.e. competition parameters), γ is the vaccine efficacy against VT carriage, *λ*_*Vi*_*(t) *and *λ*_*Ni*_*(t) *represent the age-specific force of infection for, respectively, VT and NVT pneumococci at time t.

### A.2. Mixing pattern

**Figure 9:**
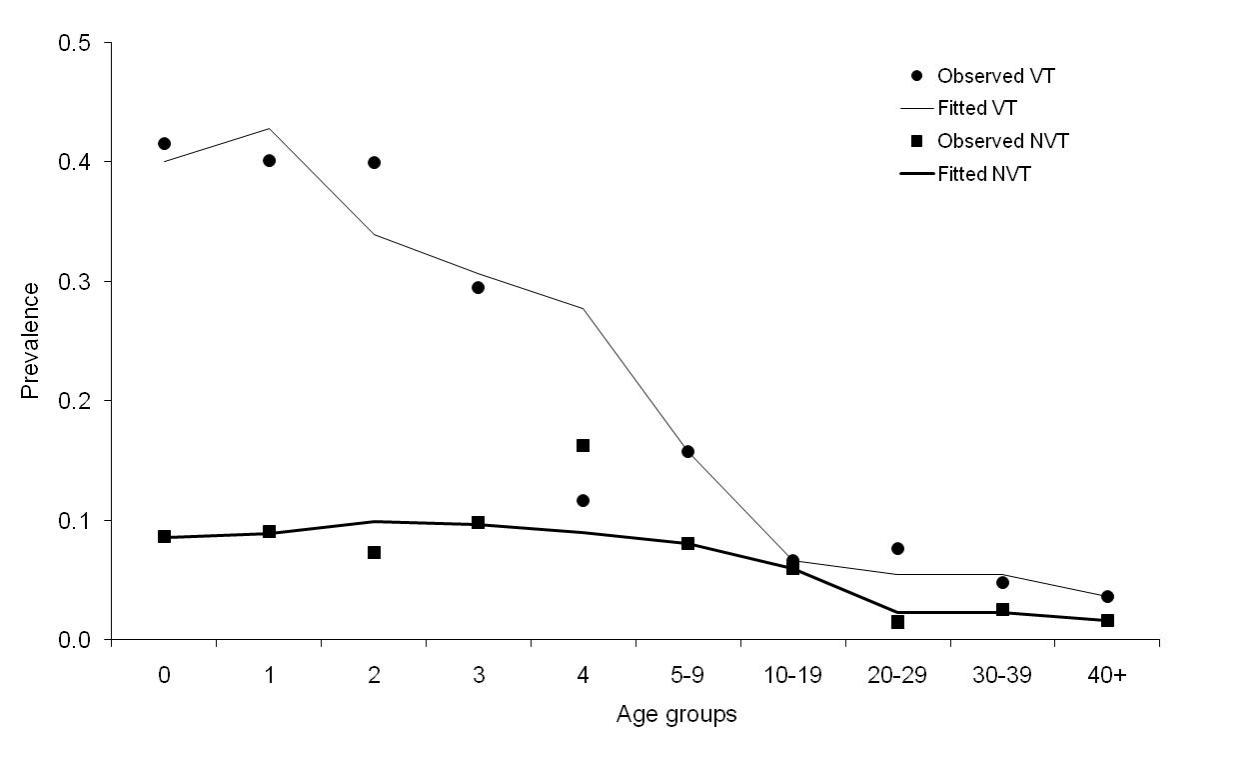
**Observed and estimated prevalence of *Streptococcus pneumoniae *carriage**.

Given values of the pneumococcal transmission parameters (in particular the competition parameters c_V _and c_N_), the effective transmission coefficients (β_Vij _and β_Nij_) were calculated by fitting a model to the number of VT and NVT carriers in each age group at steady state (Figure 9). Matrices for VT and NVT were calculated assuming fully assortative (β^a^) and proportionate (β^p^) mixing between the six age groups. A parameter ε, the assortativeness of mixing, is introduced to define the weight that is given to each of these matrices [44]:

Hence, if ε = 1, then full weight is given to the fully assortative matrices and mixing patterns is totally assortative whereas if ε = 0 full weight is given to the proportionate matrix. A value of ε is estimated when fitting to the pre and post vaccination disease incidence data for the US.

Observed and fitted prevalence of carriage of pneumococcal vaccine and non-vaccine serotypes by age group in England and Wales are presented in Figure 9. Pneumococcal carriage data were collected as part of a longitudinal pneumococcal family study in the UK that ran from October 2001 to July 2002 [31].

## Pre-publication history

The pre-publication history for this paper can be accessed here:

http://www.biomedcentral.com/1471-2334/10/90/prepub
